# Effect of Nano-TiO_2_ Composite on the Fertilization and Fruit-Setting of Litchi

**DOI:** 10.3390/nano12234287

**Published:** 2022-12-02

**Authors:** Yue Huang, Yusi Dong, Xiaobo Ding, Zhenchen Ning, Jiyuan Shen, Houbin Chen, Zuanxian Su

**Affiliations:** 1 Guangdong Litchi Engineering Research Center, College of Horticulture, South China Agricultural University, Guangzhou 510640, China; 2 Luzhou Academy of Agricultural Sciences, Luzhou 646000, China; 3 Maoming Branch, Guangdong Laboratory for Lingnan Modern Agriculture and Science, Maoming 525000, China

**Keywords:** nano-titanium dioxide, pollination fertilization, fruit-setting, litchi, rainy period

## Abstract

Titanium dioxide nanoparticles (nTiO_2_) are widely used as fertilizers in agricultural production because they promote photosynthesis and strong adhesion. Low pollination and fertilization due to rainy weather during the litchi plant’s flowering phase result in poor fruit quality and output. nTiO_2_ would affect litchi during the flowering and fruiting stages. This study considers how nTiO_2_ affects litchi’s fruit quality and pollen viability during the flowering stage. The effects of nTiO_2_ treatment on pollen vigor, yield, and fruit quality were investigated. nTiO_2_ effectively improved the pollen germination rate and pollen tube length of litchi male flowers. The germination rate reached 22.31 ± 1.70%, and the pollen tube reached 237.66 μm in the 450 mg/L reagent-treated group. Spraying with 150 mg/L of nTiO_2_ increased the germination rate of pollen by 2.67% and 3.67% for two types of male flowers (M1 and M2) of anthesis, respectively. After nTiO_2_ spraying, the fruit set rates of ‘Guiwei’ and ‘Nomici’ were 46.68% and 30.33%, respectively, higher than those of the boric acid treatment group and the control group. The edibility rate, titration calculation, and vitamin C of nTiO_2_ treatment were significantly higher than those of the control. The nTiO_2_-treated litchi fruit was more vividly colored. Meanwhile, the adhesion of nTiO_2_ to leaves was effectively optimized by using ATP and BCS to form nTiO_2_ carriers and configuring nTiO_2_ complex reagents. These results set the foundation for future applications of titanium dioxide nanoparticles as fertilizers for agriculture and guide their application to flowers and fruits.

## 1. Introduction

Nanotechnology is a breakthrough in agriculture and can potentially improve fertilizer utilization and crop yield [1,2]. The most commonly used nanoparticles in production are nZnO [3,4], nTiO_2_ [5], nAu [6], nAg [7], nCeO_2_ [8,9], nCu [10], etc. Nanoclay-attapulgite (Attapulgite—ATP) has a nanorod structure and a strong adsorption capacity. After uniform dispersion by irradiation technology, the reagent is more uniformly encapsulated [11,12]. Biomineralized silica with large specific surface area, porosity, and surface electronegativity makes up biochar-based (Biochars—BCS). As an excellent carrier for foliar fertilizer, it is applicable [13]. To create nanocomposite reagents, these two compounds can be combined as carriers. The size of nanoparticles is generally limited to less than 100 nm. They are different from macroscopic bulk structures and have small size effects, surface effects, quantum size effects, and macroscopic quantum tunneling effects [14]. Nanomaterials are often combined with carriers with slow release and degradable embedding. It can reduce volatility and extend the retention of substances, thereby maintaining the stability of nanomaterials during absorption. Moreover, nanomaterials improve the bioavailability and antioxidant capacity or prevent deactivation and harmful reactions between substance molecules due to interactions [15,16].

Nanosized anatase titanium dioxide (nTiO_2_) has a potential development capacity for agriculture. With the urgent need to meet the growing demand for nanoproducts, it has the potential to significantly increase agricultural productivity and efficiency at a lower cost and with less wasteful results [17].

nTiO_2_ is one of the most widely produced and used nanomaterials. The nTiO_2_ of anatase has exhibited high photocatalytic activity, and TiO_2_ and diatomite are low-cost, non-toxic, harmless, and stable [18]. nTiO_2_ can act as a protective layer by forming a consistent film that can absorb oxygen with a specified sensitivity [19]. TiO_2_ films are also widely used as photoelectrodes to prepare dye-sensitized solar cell devices [20]. nTiO_2_ is readily absorbed and collected by crops due to its small particle size and large specific surface area and impacts agricultural productivity and quality [21,22]. The high photoreactivity of nTiO_2_ may affect the crop’s photosynthesis and metabolism of sugars, amino acids, and fatty acids [23,24]. The crop can convert the light energy absorbed by photosynthesis into some chemical energy and then release it for growth and development [25]. Nanosized particles are more stable than large particles because they are less likely to be washed away by rainwater or to settle in the atmosphere naturally [26]. On this basis, nTiO_2_ is also usually loaded or mixed with a well-dispersed carrier material to achieve modification optimization. However, the research on the regulation of nTiO_2_ on fruit tree flowering and fruiting is still in the initial stage, and the effects on fruit tree development still need to be clarified.

Litchi (*Litchi chinensis* Sonn.) is an important tropical and subtropical fruit with a distinct flavor. Litchi is cultivated in over 20 countries between 17° N and 26° N latitudes [27], where it is an indispensable part of local economies. A suitable environment (75–80% temperature, sunny days, and light) is necessary for successful pollination and fertilization. Nevertheless, the flowering and fruiting period of litchi in southern China is mainly from March to June, which coincides with the rainy season. Artificial pollination and suitable methods are usually applied to increase fruit set and yield [28,29]. Previous work has found that boron and putrescine can improve pollen viability [30]. However, only a tiny portion of the reagents mentioned above enter the water bodies, soil, and air through rainwater drenching, washing, and evaporation, resulting in severe environmental pollution. Thus, finding a reagent that facilitates flowering time to improve fruit sets is significant.

This study aimed to investigate the effects of the nTiO_2_ and ATP- BCS/nTiO_2_ composite on the pollen vigor and fruit-setting of litchi. SEM observed the microstructure of the composite. The pollen germination rate, pollen tube length, and fruit set rates of litchi subjected to different treatments were determined. Furthermore, the edibility rate, titration calculation, vitamin c, and other fruit qualities were also evaluated. This study provided valuable information on the use of nTiO_2_ for agriculture and environmental applications during the flowering and fruiting period.

## 2. Materials and Methods

### 2.1. Experimental Site and Materials

The experiment investigated litchi’s observation of ‘Guiwei’, ‘Shuidong’, and ‘Nomici’ between March and June for two consecutive years of 2019 and 2020. The studies were conducted in two litchi orchards located in South China Agricultural University (SCAU) Main Campus Teaching & Research Base (23.164° N, 113.366° E), Guangzhou City, Guangdong province, and Luzhou Academy of Agricultural Sciences (28.889° N, 105.443° E), Sichuan Province. 

Anatase phase hydrophilic nanometer titanium dioxide (nTiO_2_) was purchased from Shanghai McLean Biochemical Technology Co., Ltd. (Shanghai, China). The appropriate 1–10 nm of nTiO_2_ was dissolved in a beaker. Ultrasonic dispersion was performed using the ultrasonic vibration function of the ultrasonic cleaner. Ultrasound was performed at 60 W, 30 kHz, and at room temperature for 30 min.

Attapulgite (ATP) was purchased from Anhui Mingmei Mineral and Chemical Co., Ltd. (Anhui, China).The attapulgite with the size of 300 mesh was modified by high-energy electron beam (HEEB) irradiation technology. The morphology of attapulgite with different irradiation doses was observed by a scanning electron microscope. The attapulgites with uniform size and structure were selected. Straw-ash-based biochar and biosilica (BCS) were collected from the paddy field of SCAU (10 March 2019). For carbonization, rice stalks were heated to high temperatures, organized, and ground into powder.

nTiO_2_, ATP, and BCS were weighed in the mass ratio of W_nTiO2_:W_ATP_:W_BCS_ = 40:8:1, respectively. ATP and BCS were diluted with a small amount of water and shaken in the ultrasonic machine for 10 min. nTiO_2_ was added and shaken for 10 min to prepare the compound reagent.

### 2.2. Design of Experiment and Field Trials

#### 2.2.1. Reagent Concentration Selection

Pollen viability was determined using pollen from the ‘Shuidong’ variety of litchi at low (16 °C to 18 °C) and average germination temperatures (24 °C to 28 °C). The nTiO_2_ solution concentration gradient was set at 0, 300, 600, and 900 mg/L. The boric acid solution concentration gradient was set at 0, 0.1, 0.2, and 0.3%. The putrescine solution concentration gradient was set to 0, 20, 40, and 60 mg/L. nTiO_2_ and its compound reagents concentration range.

#### 2.2.2. Spraying Reagent

nTiO_2_ compound reagent was sprayed on litchi flowers at the bud stage and then washed with simulated rainwater. The method of simulating rainwater scouring was to spread the same amount of moisture to simulate rainwater when the leaves were free of water after spraying the nanocomposite reagent for 1 h. Concerning the characteristics of spring rainwater in south China, raindrop diameter was mainly distributed in 0.1–5.5 mm, and rainwater was 8–10 mm [31,32].

### 2.3. Pollen Vigor

Litchi has three functionally unisexual flowers: male, female, and pseudohermaphroditic. It blooms in three waves, two male waves (M1 and M2) and one female wave (M3) [33]. The flowers at M1 were treated with titanium dioxide nanocomposite reagent. The pollen of the two male waves (M1 and M2) was collected. The female flowers received the nTiO_2_ treatment at the opening time, while the male flowers after the female flowers were M3 [34,35]. The blooming litchi male flowers were collected and dried. The pollen was collected. Regarding the pollen culture procedure, the base medium was 10.0% sucrose + 2.0% Agar to prepare different concentrations of complex reagent medium.

The Petri dishes of pollen were placed under a 10-fold positive fluorescence microscope to analyze the germination rate (pollen tube length greater than or equal to 2 times pollen grain diameter is considered effective germination) and pollen tube length (μm). The number of germinated pollen and the pollen germination rate were calculated according to the following equation:

Pollen germination rate (%) = number of germinated pollen/total pollen × 100%

Axio Imager D2 was used to monitor pollen tube viability and measure pollen tube length. Ten pollen tubes were measured randomly in each visual area, and the average length of the pollen tube was calculated. Three visual areas (about 100 pollen in each field) were observed in each petri dish.

### 2.4. Fruit Rate and Fruit Quality

The fruit rate from the litchi plantlets was estimated by counting the number of fruit at seven-day intervals from the beginning of the fruit set until fruit maturing.

The single fruit weight and edible rate of fruit soluble solids were measured by a Portable Brix Meter and titratable acids were determined by sodium hydroxide titration. Total sugars and vitamin C were determined by HPLC [36]. Fruit color was measured using a Minolta CR-400 automatic colorimeter made in Japan.

### 2.5. Characterization of nTiO_2_ Composite Reagent

The Zeiss EVO MA15 scanning electron microscope was used to observe the microscopic morphology of titanium dioxide nanoparticles, intaglio, and biochar with a resolution of 3 nm and an X-ray energy spectrum. Surface microscopic characterization was performed using a 0.6 nm resolution with an X-ray spectrum acceleration voltage of 10.00 kV, a secondary electron image signal, and a working distance of 4.0 mm [37]. A Fourier transform infrared spectroscopy pattern (FTIR) was carried out on a VERTEX 70v. It was studied in the frequency range of 400–4000 cm^−1^.

### 2.6. Statistical Analysis

The trial was carried out based on a randomized block design, repeated three times. Excel 2013 and SPSS 19.0 were used to statistically evaluate data. The T-test and ANOVA were used to analyze the significance of the results. Multiple comparisons were also tested by Duncan’s new repolarization method at the *p* < 0.05 level. 

## 3. Results and Discussion

### 3.1. Pollen Viability Was Improved by nTiO_2_

**Figure 1:**
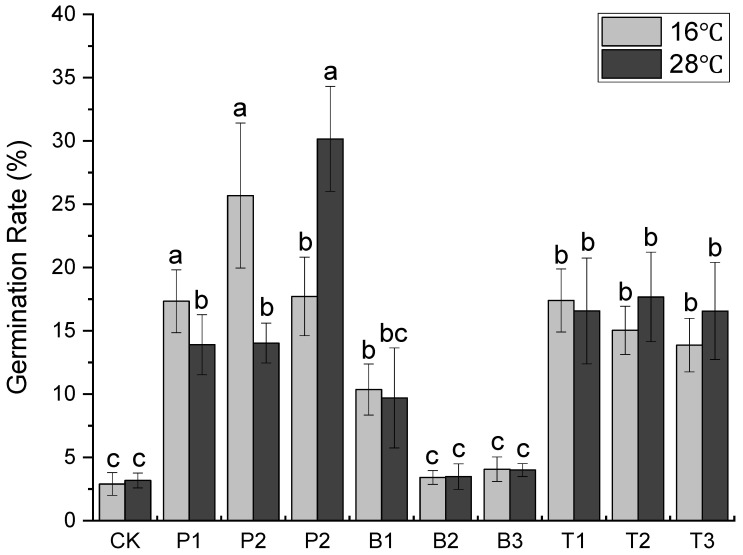
Germination rate of litchi pollen in different concentrations of reagents. CK: Clearwater P1, P2, P3: putrescine 20 mg/L, 40 mg/L, 60 mg/L. B1, B2, B3: boric acid 0.1%, 0.2%, 0.3%. T1, T2, T3: Nano-titanium dioxide 300 mg/L, 600 mg/L, 900 mg/L. Different letters indicate statistical differences between treatments (*p* ≤ 0.05).

Putrescine can enhance the activity of pollen and stigma receptivity. As shown in Figure 1, putrescine significantly increased the germination rate of ‘Shuidong’ litchi. The germination rates of Putrescine treatments ranged from 13 to 30%, which was significantly higher than the control. The higher the temperature, the higher the effective concentration of Putrescine required. Except for the germination rate of 0.1 percent of 10.36% and 8.73% of boric acid at 16 °C and 28 °C, the other two concentrations and CK did not increase significantly. Previous studies have shown that boric acid can increase pollen germination [38]. The effect was better at 0.1% boric acid and low temperatures. At both temperatures, nTiO_2_ had a substantial impact on all three concentrations. However, it was lower at 16 °C, at 60 mg/L, than at 28 °C. Compared to the control, nTiO_2_ significantly increased the pollen germination rate, which remained between 13.87 and 17.67% at both 16 and 28 °C. This is connected to earlier discoveries that nTiO_2_ can improve the growth of soybean plants, increase water and oxygen uptake by roots, and increase the oxidative stress capacity and stress resistance in soybean [39,40,41]. This finding suggests that nTiO_2_ is relatively stable and has a similar impact on pollen germination. However, putrescine works best for germination rate under specific conditions. Nevertheless, putrescine has highly toxic limits [42] and is not stable in use. The boric acid only works at 0.1%. The non-toxic nTiO_2_ sprouts more readily at various temperatures and concentrations. Thus, nTiO_2_ is considered to have more application advantages.

**Figure 2:**
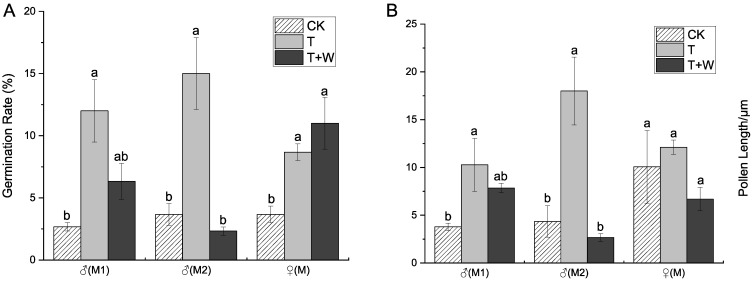
Pollen germination rate (**A**) and tube length (**B**) of different batches after nTiO_2_ treatment. M1: first batch of male flowers. M2: second batch of male flowers. M3: female flower. CK: 0 mg/L nTiO_2_. T: 150 mg/L nTiO_2_. T + R: 150 mg/L nTiO_2_ + Water Brush. Different letters indicate statistical differences between treatments (*p* ≤ 0.05).

Litchi flowers come into the anthesis in three separate waves, associated with environmental factors or the nutritional status of the branches. The experiment was repeated in both fluctuations to determine whether nTiO_2_ affects pollen promotion in different states. As shown in Figure 2A, the pollen germination rate of T from stage M1 to stage M3 was 8.67 ± 0.67% to 15.00 ± 2.89%, significantly higher than that of the control group. T + W treatment did not significantly increase the germination rate of pollen at stages M1 and M2, with the highest rate (11.00 ± 2.08%) at stage M3. The pollen viability of male flowers was typically low, but the nTiO_2_ reagent could increase the pollen viability. The higher M3 germination rate could be attributed to the removal of male flowers at the M1 stage, which reduced the effect of rain on composting flowers. The pollen tube length of pollen (Figure 2B) of the first stage (M1) using nTiO_2_ was 10.27 ± 2.76 μm, significantly higher than the 3.78 ± 0.40 μm of the control group. The length of the pollen tube after simulated rainwater scouring was not significant. The second stage batch of male flowers (M2) had a pollen tube length of 17.99 ± 3.54 μm, which was significantly longer than the control batch’s pollen tube length of 4.36 ± 1.65 μm. Moreover, the pollen tube length after the nTiO_2_ and the simulated rainwater was not significantly increased. With a specific concentration, nTiO_2_ can effectively improve the pollen tube length of male flowers. According to the results, the spraying of nTiO_2_ at the bud stage had a significant effect on pollen viability. At the same time, the outcome of the T + W treatment was not significantly different from that of the control group. It might be because scouring dilutes the concentration of nano-reagents. A subsequent search for methods to improve nTiO_2_ adhesion and the rate of nTiO_2_ uptake in leaves is still required.

### 3.2. nTiO_2_ Increased the Yield of Litchi

**Figure 3:**
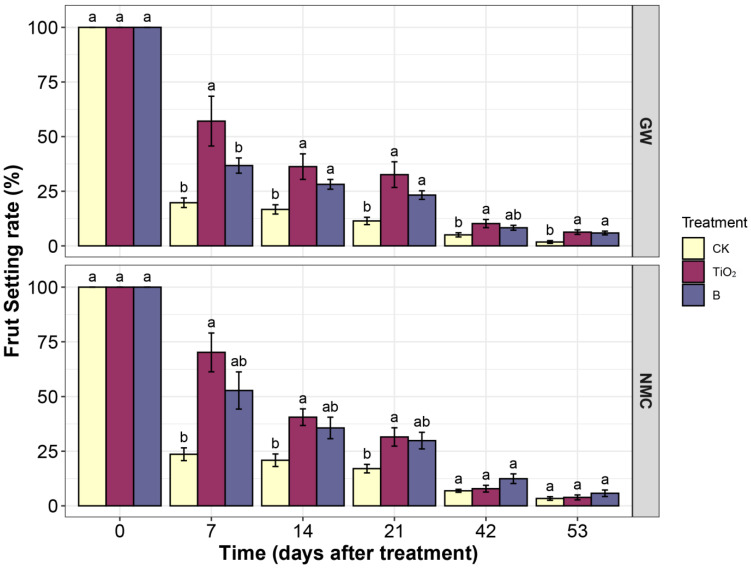
Fruit set influenced by nTiO_2_ and boric acid in litchi. GW: litchi cultivar ‘Guiwei’; NMC: litchi cultivar ‘Nuomici’. B: Boric acid. Different letters indicate statistical differences between treatments (*p* ≤ 0.05).

Figure 3 shows the dynamics of the fruit rate of ‘Guiwei’ and ‘Nuomici’. Both boric acid and nTiO_2_ improved the fruit set rate of litchi. The fruit rate of ‘Guiwei’ was recorded at its highest 7 days after nTiO_2_ treatment. nTiO_2_ increased by 37.35 ± 9.20% and 20.34 ± 7.90% compared to CK and boric acid treatment. The fruit rate was 36.27 ± 5.84% and 32.60 ± 5.88% at 14 and 21 days after the nTiO_2_ spraying treatments, which were higher than the control (16.69 ± 2.12% and 11.41 ± 1.68%). The effect on ‘Nuomici’ showed a similar trend. The fruit rate of the nTiO_2_ group was 52.79 ± 8.51%, 46.56 ± 5.92%, and 17.38 ± 0.33% at 7, 14, and 21 days after treatment, which was higher than those of the CK group of 23.61 ± 2.92%, 20.86 ± 2.87%, and 17.03 ± 1.95%, respectively. Our results reinforce the finding that the application of boric acid significantly increases the yield and quality of crops [43,44], while nTiO_2_ can improve the yield of strawberries [45,46]. The data also suggested that nTiO_2_ showed a more significant effect than boric acid. nTiO_2_ improved fruit set in both litchi varieties ‘Guiwei’ and ‘Nuomici’, with ‘Guiwei’ being more pronounced. 

**Figure 4:**
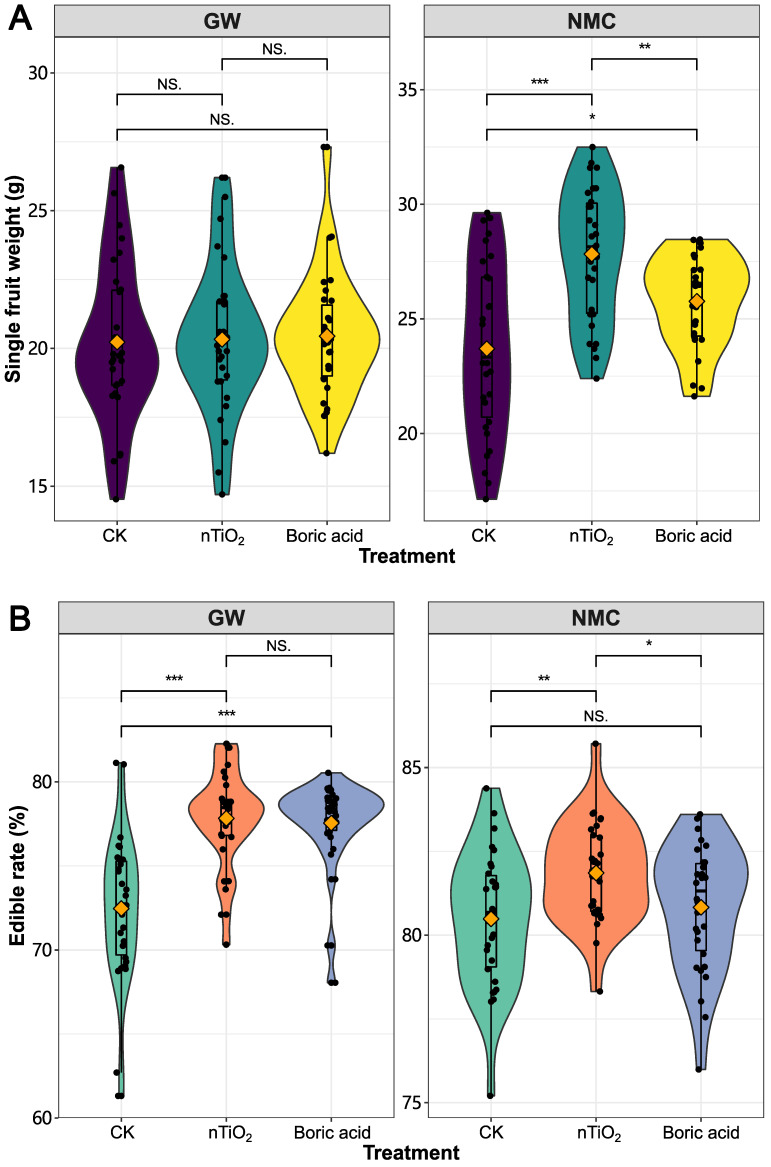
The effect of nTiO_2_ and boric acid on single fruit weight and edible rate of litchi. GW: litchi cultivar ‘Guiwei’. NMC: litchi cultivar ‘Nuomici’. NS, no significance differences; *, *p* ≤ 0.05; **, *p* ≤ 0.01; ***, *p* ≤ 0.001.

The data revealed that the single fruit weight of ‘Nomici’, and the edible rate of ‘Guiwei’ and ‘Nomici’ were significantly higher in nTiO_2_ treatment (Figure 4). The single fruit weight of ‘Nuomici’ after the nTiO_2_ treatment was markedly higher than those of the control and boric acid treatment groups, up to 27.83 ± 0.52 g. Comparing the edible rate of the two species, the TiO_2_ treatment was significantly higher than those of the control in both varieties. Especially in the array ‘Nuomici’ than in the boric acid treatment group, it was 81.9 ± 0.27% and 77.93 ± 5.23% in both.

### 3.3. nTiO_2_ Improved the Fruit Quality of Litchi

In addition to the improvement of fruit set rate, nTiO_2_ also has a promotion effect on the enhancement of fruit nutrient accumulation. The soluble fruit index of ‘Guiwei’ pollinated with nTiO_2_ treatment was 19.4%, significantly higher than those of boric acid and the control. Vitamin C content showed that the nTiO_2_ treatment was more significant than the control, at 0.91 ± 0.03 mg/g, while the control was only 0.72 ± 0.01 mg/g. The soluble solids of ‘Nomici’ were 19.10 ± 0.12%, titratable acid 0.19 ± 0.00%, and vitamin C content 0.96 ± 0.00 mg/g. They were considerably higher than the control and boric acid treatment, while the solid-to-acid ratio was significantly lower than that of the control (Table 1). Treatment with a mixture of litchi pollen and boric acid resulted in no difference between the single fruit weight and soluble solids of ‘Guiwei’ and the control, and the vitamin C content was significantly higher than that of the control, whereas the solid-to-acid ratio was significantly lower. The edibility and soluble solids of ‘Nomici’ were not different from those of the control, but the vitamin C content was significantly higher than that of the control, and the solid-to-acid ratio was significantly lower than that of the control. The fruits that had been treated with nTiO_2_ were sweeter.

Applying boric acid during the fruit setting can also increase the quality of the fruit [47]. The soluble solids and solid acids of ‘Guiwei’ pollinated with nTiO_2_ were higher than those of the boric acid treatment. The single fruit weight, edibility, soluble solids, solids-to-acid ratio, and vitamin C content of ‘Nomici’ fruits pollinated by nTiO_2_ were higher. Fruit from both kinds treated with nTiO_2_ had less titratable acid than fruit treated with boric acid. nTiO_2_ treatment fruit had better flavor and texture. Nutrients during flowering affect the final litchi fruit setting and its fruit quality. The nutrient source of fruit ripening is not the early accumulation of the tree but the timely application of fertilizer during the flowering and fruit period [48]. nTiO_2_ increased nutrient levels during flowering, promoting fruit quality improvement. As previously discovered, boric acid benefits the growth, fruit, and oil yield of olive quality [49], and similarly, nTiO_2_ has a similar effect on pear [50]. Both reagents can improve the single fruit weight of ‘Nuomici’, and the nTiO_2_ treatment was more remarkable than the boric acid. As a result, it is visible that litchi flower powder binds to nTiO_2_ or B + ZuSO_4_ + CaCl_2_ + Sucrose aqueous solution to improve the effect of ‘Guiwei’, and the impact of nTiO_2_ treatment is relatively stable.

nTiO_2_ can improve the degree of fruit color of litchi. The skin color of ‘Guiwei’ and ‘Nuomoci’ litchi was determined experimentally (Table 2). The result showed ‘Guiwei’ peel color nTiO_2_ and boric acid treatment a* value of about 41 c* value of about 40. Both pollination treatments are significantly higher than the control. In contrast, both the boric acid and nTiO_2_ treatment groups had L* values around 42, b values around 24, and h values around 38, which were significantly lower than those of the control. The results showed that the treated fruits were redder and brighter in color. The a* value of nTiO_2_ treatment ‘Nuomici’ fruit was about 29, which was significantly higher than those of the control and boric acid treatment, and the peel was red, but the b* value was about 24 and the h value was about 40, which was significantly lower than those of the control. The boric acid treatment was more lustrous than the nTiO_2_ treatment, but the color was orange and more mature.

‘Guiwei’ and ‘Nuomici’ litchi ripe fruit’s peel color was general red beltless blue col-or at the most appropriate time. Table 2 indicates that the pollination treatment of nTiO_2_ and boric acid could improve the sensory quality of the litchi appearance and the color of the litchi peel. The results are the same as previous studies that concluded that boric acid could maintain higher fruit hardness, total sugars, and total phenols, which extends the storage life of the fruit [51]. However, nTiO_2_ was brighter and better than the litchi fruits treated with boric acid. nTiO_2_ can degrade ethylene to delay the post-ripening of bananas [52], and the same may also exist for the preservation of litchi. The results also indicated that the fruits treated with nTiO_2_ were relatively red and of better quality.

In this study, nTiO_2_ may stabilize the early fruit set of two litchi varieties, ‘Guiwei’ and ‘Nuomici’. The effect of nTiO_2_ on the fruit quality of different types of litchi may be different, and the comparison showed that it had a more significant impact on ‘Nuomici’. Indicators such as edible rate had a more evident and positive impact on ‘Nuomici’. The final fruit increase was set by applying nTiO_2_ during the buds-welling stage and before blooming. It could be explained by its constructive effect on pollen viability and tube elongation [46]. It also has the effect of decomposing the ethylene in the fruit and delaying the preservation of freshness so that the fruit color remains fresh [53].

### 3.4. nTiO_2_ Composite Reagent

#### 3.4.1. Structure Diagram and SEM Analysis

**Figure 5:**
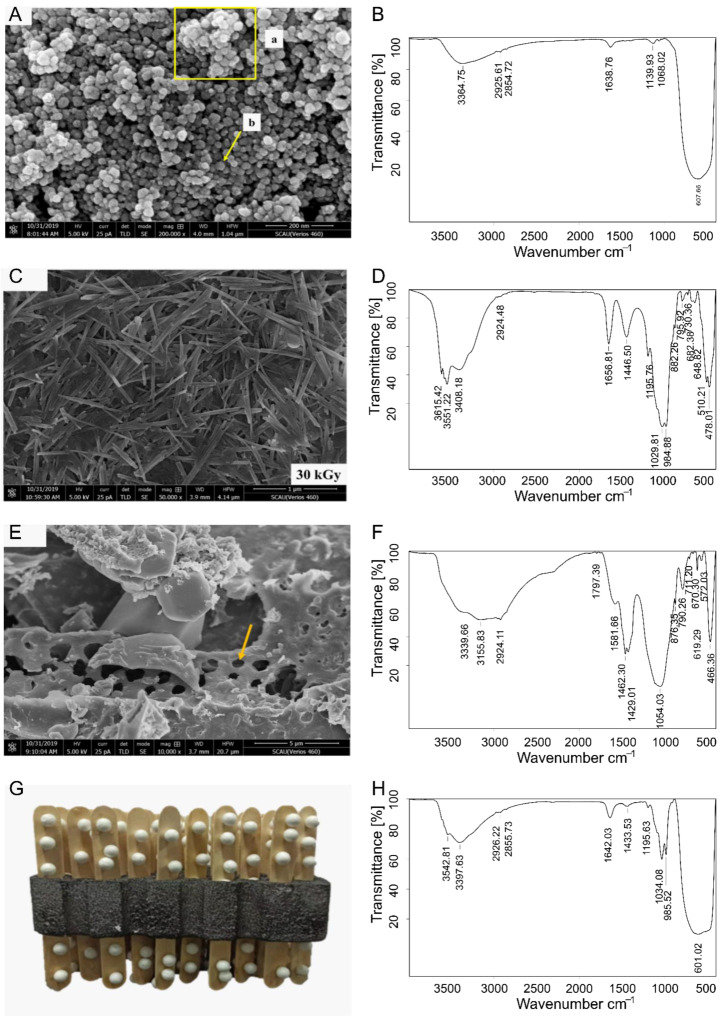
nTiO_2_ composite reagent. (**A**): SEM image of P25 nTiO_2_: a: agglomeration, b: dispersion; (**B**): FTIR spectrum of nTiO_2_; (**C**): 30 kGy radiation ATP SEM image; (**D**): FTIR spectrum of ATP; (**E**): BCS SEM image; Arrow indicates the microporous structure of BCS at 10,000× magnification under SEM; (**F**): FTIR spectrum of BCS. (**G**): Ideal structure of composite reagent; (**H**): FTIR spectrum of nTiO_2_ composite reagent.

This experiment designed a new nTiO_2_ composite reagent, and the exemplary structure diagram is shown in Figure 5A. nTiO_2_ is a good fertilizer for field application. However, the problem of fertilizer being washed away by rain in the field has always existed. Due to the inherent lotus effect on the foliage of crops [54], most foliar fertilizers fall off the foliage during the spraying process. Through rainwater flushing and irrigation, fertilizers are discharged into the soil, rivers, and other environmental media. It seriously pollutes the environment and wastes human resources [55]. Therefore, nTiO_2_ complexes need to be studied to increase the adhesion of nTiO_2_ and reduce the adverse effects of rainwater scouring.

This model can increase the specific surface area of titanium dioxide nanoparticles and prevent them from adhering together. SEM was used to characterize the nTiO_2_ composite reagent. The study presented the SEM images of the pure nTiO_2_ with a 200,000-fold magnification (Figure 5B). The pure nTiO_2_ was between 1 and 5 nm, relatively uniform. The surface was close to round. The smaller the particle size is, the more pronounced the mutual adsorption of the material is [56]. The nTiO_2_ without ultrasonic dispersion had some particle agglomeration (Figure 5A, a). nTiO_2_ particles are prone to form agglomerations when they are not modified. It is speculated that agglomeration will lead to excessive concentration and affect absorption [57,58]. There are ultrasonically dispersed nTiO_2_ that can be well broadcast (Figure 5A, b). The attapulgite modified by irradiation (Figure 5C) showed that the beam distribution after dose treatment was more uniform. The micropore and nanopore structure of rice straw ash facilitated the dispersion of ATP by steric hindrance (Figure 5D). The micropores of rice straw ash can accommodate concavity and nTiO_2_, which are suitable as carriers of nTiO_2_. These three constitute composite reagents. Studies have shown that nanomaterials can enter plant cells by binding to carrier proteins, through ion channels, or endocytosis [59,60]. The gravitates can be more uniformly encapsulated with reagents after they are uniformly dispersed by the irradiation technique [61].

nTiO_2_ was modified by a high-energy electron beam (HEEB) to form uniformly structured nanoscale gravitates, which were uniformly crossed in the incinerated straw ash. nTiO_2_ was uniformly distributed in the gravitates by ultrasonic vibration to reduce agglomeration. Adding attapulgite and straw-ash-modified nanocomposite reagent can make the nTiO_2_ enter between the micropores, and the attapulgite can make it evenly dispersed, avoiding the reunion [62,63]. Straw ash with a microporous structure was added to the suspension of attapulgite to enhance its dispersion performance as a naturally agglomerated nano-clay. The attapulgite and straw ash nanocomposite (ATP-BCS) were created using ultrasound. XRD (X-ray diffraction), SEM, and FTIR (Fourier Transform infrared spectroscopy) spectra showed that the attapulgite was successfully combined with straw ash through hydrogen bonding and physical interaction [64]. Figure 5B, H indicate the FTIR spectra powder solubility changes of the composite reagent and monomer, respectively. The peak at 601 cm^−1^ is attributed to Ti−O stretching [65]. For the characteristic cliff of the composite reagent whose hydroxyl component appears near 3397 cm^−1^, the intensity of the peak after adsorption significant decreases compared with that before adsorption. There is a wrapping effect after adsorption to the surface of TiO_2_ NPs, thus reducing the hydroxyl group on the surface of TiO_2_ NPs. The new stretch at 1034 cm^−1^ can be assigned to ATP, the peak produced by BCS. It has been found that ATP and BCS constitute carriers with some adhesion ability to nitrogen fertilizer. The reference method optimized the compounding reagent ratio to W_nTiO2_:W_ATP_:W_BCS_ = 40:8:1 [66,67]. Concave bump stone-grass ash complex or grass ash was added as a substrate or carrier to improve the adhesion of fertilizer and reduce environmental pollution [68]. After modifying the experiment, the composite reagent was completed by inserting BCS after the uniform adhesion of nTiO_2_ to ATP by the ultrasonic technique. The three materials combined to form an experimental nTiO_2_ composite reagent, effectively increasing the nTiO_2_ absorption capacity and improving its adhesion to leaves. 

#### 3.4.2. Adhesion of nTiO_2_ Compound Reagents

**Figure 6:**
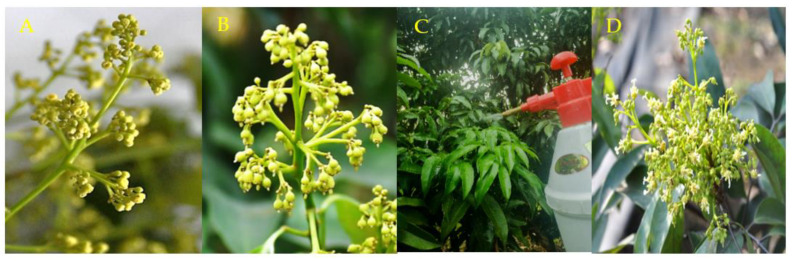
Flower bud nTiO_2_ composite reagent and simulation wiper treatment. (**A**): Female flower bud; (**B**): male flower bud; (**C**): flower bud spray nTiO_2_ composite reagent; (**D**): artificial pollination simulation of rainwater.

**Figure 7:**
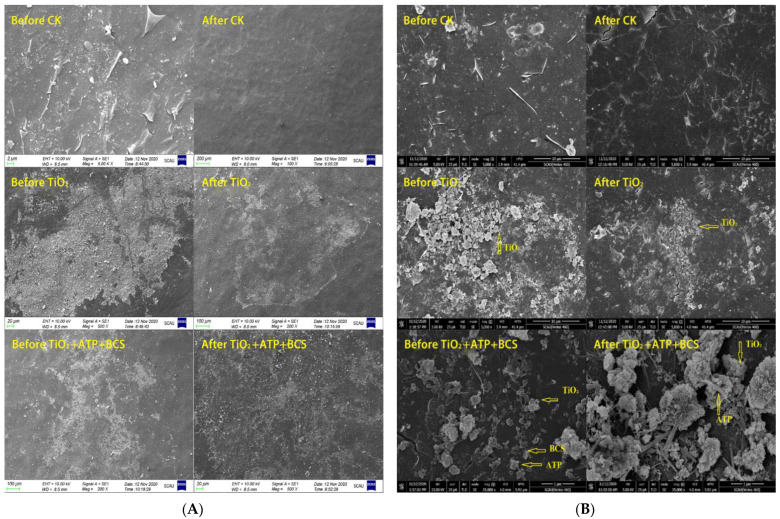
The blade of the nTiO_2_ composite reagent distribution. (**A**): 500× or less; (**B**): 5000× or more; Before CK: Before CK spun; After CK: After CK spun; Before TiO_2_: Before nTiO_2_ spun; After TiO_2_: After nTiO_2_ spun; Before TiO_2_ + ATP + BCS: Before nTiO_2_ composite reagent spun; After TiO_2_ + ATP + BCS: After nTiO_2_ composite reagent spun.

The nTiO_2_ composite reagent improved the adhesion of the monomer. Through a simulated wiper experiment [69,70], an analysis experiment was developed to explore the adhesion of nTiO_2_. At the bud stage, pistil and stamens were sprayed with nTiO_2_ compound reagent (Figure 6A,B) and washed with simulated rainwater (Figure 6C). After flushing, artificial pollination was performed at the pistil stage (Figure 6D). As shown in Figure 7, impurities were significantly reduced after simulated rainfall. In the control group, the leaf impurities were washed away, showing that the simulated rainwater effectively flushed the material. Residual nTiO_2_ was found in the treatment group sprayed with monomeric nTiO_2_ and nTiO_2_ compound reagents. Among them, residuals were observed in the nTiO_2_ compound reagent with more significant residues. The composite reagent has a more striking effect. The adhesion of the nTiO_2_ composite reagent can make bacteria effectively adhere to plant roots [71]. 

The study indicates that introducing iron oxides in biochar-clay composites can reduce the tendency of iron oxide agglomeration [72]. Fertilizers of standard block sizes do not have good adhesion properties. The leaf surface fertilizer may be washed away in the rain. nTiO_2_’s adhesion makes it less likely to be washed away by rain after washing [73]. It also enabled the monomers to adhere well to the uniformly sized gravels modified by the radiation technique, resulting in a more uniform distribution and less agglomeration. The test proves that the nTiO_2_ compound reagent improves the adhesion and can effectively play the role of fertilizer. However, it still needs to be explored whether the composite reagent masks part of the nTiO_2_ and whether the monomer works better in the absence of adversity.

#### 3.4.3. Yield Related Attributes Influenced by Different Reagents

nTiO_2_ complex reagent can improve the quality of litchi fruits. The results in Table 3 show that hand pollination with nTiO_2_ complex reagent resulted in a fruit weight of 23.4 ± 3.50 g for ‘Guiwei’, which was significantly higher than hand pollination alone. Fruit peel and fruit flesh weight were increased considerably under pure nTiO_2_ treatment. The fruit flesh weight was 19.2 ± 0.9 g, and the peel weight was 1.5 ± 0.0 g. The cells of the pericarp and mesocarp were enlarged, thus affecting the pericarp weight and fruit weight [74]. The increase in peel weight is beneficial to reduce the late fruit cracking of litchi [75]. Especially for ‘Nuomici’, ‘Sanyuehong’ and other varieties of litchi with thin skin can effectively improve productivity.

There were no significant differences in fruit set rate among the pollination treatments. Regarding fruit edible rate, artificial pollination without reagent was 82.3 ± 2.7%, significantly higher than that of boric acid artificial pollination. The results of soluble solids, boric acid treatment, and nTiO_2_ complex reagents were significantly higher than that of the artificial pollination. Among them, the compound reagent increased the TTS content the highest and had the most significant effect.

## 4. Conclusions

The study was undertaken to fill the gaps in the possible changes in the generative and reproductive stages of fruit growth after exposure to nTiO_2_. We discovered that the nTiO_2_-treated variant improved pollen vitality and the fruit quantity of litchi. The results showed that nTiO_2_ promotes reproductive processes, especially the rate of pollen germination and pollen tube length at the tested dosages. The nTiO_2_ provided unexpected early plant maturation with physiological indices. To determine the effect of nTiO_2_ on the reproductive stage, we continuously observed the state of litchi fruits after nTiO_2_ treatment. In contrast, nTiO_2_ treated and better reflected the litchi physiological parameters with relatively sound quantitative and nutritional effects. This subsequently improved the litchi’s capacity for pollination, fertilization, and fruit setting. nTiO_2_ had a significant impact on fruit set and quality, notably increasing fruit weight, fruit color, and TSS. The edibility rate, titration calculation, and vitamin C of nTiO_2_ treatment significantly increased. The nTiO_2_-treated litchi fruit was more vividly colored. Meanwhile, we successfully green-synthesized nTiO_2_ composite reagent using ATP and BCS. The synthesized nTiO_2_ composite reagent was adhesion-rich and smaller in size. The synthesized nTiO_2_ composite reagent showed its potential for fruit development. However, experimental verification is required for its safe utilization. Our findings reveal the potential of nTiO_2_ application to promote the quality of fruits with pollen viability and fruit quality, which is helpful for developing high-quality nano-enabled agriculture in the future. Further investigation is needed regarding the appropriate concentration required and the way of absorption and utilization in the plant.

## Figures and Tables

**Table 1:** Fruit quality influenced by nTiO_2_ and boric acid in litchi.

Variety	Treatment	TTS (%)	TA (%)	Vc (mg/g)	TSS/TA
GW	CK	18.87 ± 0.15b	0.15 ± 0.00b	0.72 ± 0.01b	124.29 ± 2.18a
	nTiO_2_	19.40 ± 0.00a	0.15 ± 0.00b	0.91 ± 0.03a	126.05 ± 3.16a
	Boric acid	18.93 ± 0.03b	0.17 ± 0.00a	0.89 ± 0.00a	110.14 ± 1.36b
NMC	CK	17.93 ± 0.26b	0.14 ± 0.00b	0.73 ± 0.00c	125.53 ± 2.75a
	nTiO_2_	19.10 ± 0.12a	0.19 ± 0.00a	0.96 ± 0.00a	101.81 ± 0.61b
	Boric acid	17.70 ± 0.46b	0.20 ± 0.00a	0.89 ± 0.02b	88.14 ± 3.03c

TSS: Total soluble solid content. TA: Titratable acidity. Vc: Vitamin C. TSS/TA: TSS-to-TA ratio. GW: ‘Guiwei’. NMC: ‘Nuomici’. Different letters indicate statistical differences between treatments (*p* ≤ 0.05).

**Table 2:** ‘Guiwei’ and ‘Nuomici’ maturity peel color after the pollination treatment.

Variety	Treatment	L*	a*	b*	c*	h
GW	CK	45.74 ± 0.99a	22.31 ± 1.54b	28.36 ± 0.67a	36.72 ± 0.60b	52.39 ± 2.47a
	TiO_2_	41.72 ± 0.90b	30.7 ± 0.72a	24.09 ± 0.72b	39.22 ± 0.45a	38.17 ± 1.34b
	Boric acid	42.25 ± 0.56b	31.81 ± 0.51a	24.14 ± 0.45b	40.02 ± 0.35a	37.23 ± 0.85b
NMC	CK	42.39 ± 0.76ab	25.21 ± 0.93b	26.53 ± 0.41a	36.81 ± 0.46a	46.73 ± 1.45a
	TiO_2_	40.50 ± 0.78b	29.18 ± 0.97a	24.17 ± 0.56b	38.13 ± 0.54a	39.87 ± 1.53b
	Boric acid	44.07 ± 0.76a	26.71 ± 1.20ab	25.97 ± 0.52a	37.59 ± 0.61a	44.62 ± 1.81a

L* denotes luminosity, which is equivalent to brightness; a* denotes the range from magenta to green; b* denotes the range from yellow to blue; c* denotes the degree of color saturation or purity; h denotes the hue angle. GW: ‘Guiwei’. NMC: ‘Nuomici’. Different letters indicate statistical differences between treatments (*p* ≤ 0.05).

**Table 3:** Artificial pollination of ‘Guiwei’ fruit quality under different reagents.

Treatment	Fruit Weight (g)	Fruit Peel Weight (g)	Fruit Flesh Weight (g)	Seed Weight (g)	Fruit Transverse Diameter (mm)	Fruit Longitudinal Diameter (mm)	Fruit Set Rate (%)	Stenospermocarpic Rate (%)	Edible Rate (%)	TTS (%)
Artificial pollination	19.5 ± 1.4b	2.4 ± 0.1b	16.2 ± 0.6b	1.0 ± 0.3a	32.6 ± 1.0ab	32.4 ± 1.0a	3.47 ± 0.9%a	66.7 ± 8.8%a	82.3 ± 2.7%a	16.87 ± 0.13%b
Boric acid	16.4 ± 1.3c	2.5 ± 0.1ab	12.7 ± 0.9c	1.6 ± 0.2a	29.6 ± 1.1b	28.8 ± 1.0b	1.16 ± 0.3%a	30.0 ± 16.5%b	74.3 ± 1.8%b	17.9 ± 0.2%a
nTiO_2_ Compound reagent	23.4 ± 3.5a	2.7 ± 0.2a	19.2 ± 0.9a	1.5 ± 0.0a	33.9 ± 1.3a	33.8 ± 1.3a	1.51 ± 1.1%a	55.0 ± 5.0%a	81.5 ± 1.5%a	17.9 ± 0.3%a

The fruit quality results of the compound reagent were analyzed. Artificial pollination with the addition of nano-compound reagent improved the fruit set rate and quality of litchi while enhancing the single fruit weight and fruit edibility of ‘Guiwei’. In both fruit weight and TTS, the nTiO_2_ complex reagent can play an enhancing role. It also demonstrated that the composite reagent had adhesion and could reduce the effect of rain flushing while increasing litchi yield and stability. Different letters indicate statistical differences between treatments (*p* ≤ 0.05).

## Data Availability

Not applicable.
